# Detection of Bitterness in Vitamins Is Mediated by the Activation of Bitter Taste Receptors

**DOI:** 10.3390/nu14194141

**Published:** 2022-10-05

**Authors:** Thomas Delompré, Christine Belloir, Christophe Martin, Christian Salles, Loïc Briand

**Affiliations:** CSGA (Centre des Sciences du Goût et de l’Alimentation), CNRS, INRAE, Institut Agro, Université de Bourgogne-Franche Comté, 21000 Dijon, France

**Keywords:** vitamins, bitter taste, TAS2R, cellular assay, sensory analysis

## Abstract

Vitamins are known to generate bitterness, which may contribute to an off-taste or aftertaste for some nutritional supplements. This negative sensation can lead to a reduction in their consumption. Little is known about the bitter taste threshold and taste sensing system for the bitter taste detection of vitamins. To better understand the mechanisms involved in bitterness perception, we combined taste receptor functional assays and sensory analysis. In humans, bitter taste detection is mediated by 25 G-protein-coupled receptors belonging to the TAS2R family. First, we studied the bitterness of thirteen vitamins using a cellular-based functional taste receptor assay. We found four vitamins that can stimulate one or more TAS2Rs. For each positive molecule–receptor combination, we tested seven increasing concentrations to determine the half-maximal effective concentration (EC_50_) and the cellular bitter taste threshold. Second, we measured the bitter taste detection threshold for four vitamins that exhibit a strong bitter taste using a combination of ascending series and sensory difference tests. A combination of sensory and biological data can provide useful results that explain the perception of vitamin bitterness and its real contribution to the off-taste of nutritional supplements.

## 1. Introduction

Bitterness is an indicator of potential toxicity and/or possible microbial contamination, mediating an aversive response. This quality of taste is a warning and defense mechanism that provides protection against intoxication [1,2]. Many nutrient compounds, such as minerals, amino acids, and vitamins, have a strong bitter taste [3,4].

There are thirteen known vitamins belonging to two groups: the water-soluble vitamin group, including vitamin C and members of the vitamin B family (B1, B2, B3, B5, B6, B8, B9, and B12), and the fat-soluble vitamin group, composed of four vitamins (A, D, K, E). Overall, foods contain varying amounts of vitamins, which are involved in various biological processes. Therefore, all of these vitamins are essential for the correct function of the human body. They can operate as coenzymes and transporters of electrons and protons and can be involved in membrane stabilization and in some hormonal and taste functions. For example, vitamin E (in its tocopherol form) is a powerful antioxidant that limits the spread and impact of free radicals on an organism [5]. Vitamin B2 (in its riboflavin form) plays an essential role in the redox reactions of many metabolic pathways [6].

Like many natural compounds, vitamins have varying taste properties according to their structure, which influence the organoleptic qualities of some foods. While vitamin C is described as sour, many other vitamins, mainly B vitamins such as B1 and B3, have a strong bitter taste [3,7]. As vitamins have beneficial effects on human health, they are encountered in many pharmaceutical preparations, nutritional supplements, and food supplements. However, these vitamin-based products are poorly accepted by patients and/or consumers, which significantly reduces their health benefits [8]. This decrease in product acceptability is directly related to the presence of an off-taste, which can be attributed to the bitterness of certain vitamins [9,10].

In humans, the activation of 25 bitter taste receptors (TAS2Rs) is involved in bitter taste detection. Bitter taste receptors belong to the class A subfamily of G-protein-coupled receptors (GPCRs) [11,12]. These GPCRs are mainly expressed in the taste buds of papillae. Heterologous functional expression experiments have revealed the activation profile of 21 of the 25 TAS2Rs [13]. Some bitter taste receptors (TAS2R10, TASR14, and TASR46) are qualified as broadly tuned receptors that can be used to detect a large variety of natural and synthetic bitter compounds [13,14]. The TAS2R3, TAS2R5, TAS2R8, TAS2R13, TAS2R20, TAS2R41, and TAS2R50 receptors are narrowly tuned receptors involved in the detection of a small diversity of bitter tastants [13,15]. The TAS2R1, TAS2R4, TAS2R7, TAS2R9, TAS2R30, TAS2R31, TAS2R39, TAS2R40, and TAS2R43 receptors are referred to as moderately tuned receptors since they can be used to detect a greater or lesser number of compounds [13,16]. Two other bitter taste receptors, TAS2R16 and TAS2R38, have been identified as being involved in the detection of compounds sharing the same chemical characteristics [17]. No agonists have been identified for the TAS2R19, TAS2R42, TAS2R45, and TAS2R60 receptors, which are categorized as orphan receptors.

A few sensory studies have demonstrated the bitterness of some vitamins [7,18], which can be detected through the activation of TAS2R receptors. Indeed, it has been demonstrated that the bitterness of thiamine hydrochloride (vitamin B1) is mediated by the activation of three TAS2Rs (TAS2R1, TAS2R7, and TAS2R39) [13,19]. However, no information is available on the taste threshold and sensory sensing mechanism involved in the bitterness detection for other vitamins.

To better understand these mechanisms and evaluate the impact of vitamin use on the sensory qualities of nutritional supplements, we combined taste receptor functional assays and human sensory analysis. The three objectives of this study were to (i) determine the capacity of thirteen vitamins to activate the 25 TAS2Rs, (ii) measure the human bitter taste detection threshold in an aqueous solution for vitamins identified as bitter, and (iii) compare and correlate the bitter taste detection of vitamins with receptor activation.

## 2. Materials and Methods

### 2.1. Taste Receptor Functional Assay

#### 2.1.1. Chemical

**Figure 1:**
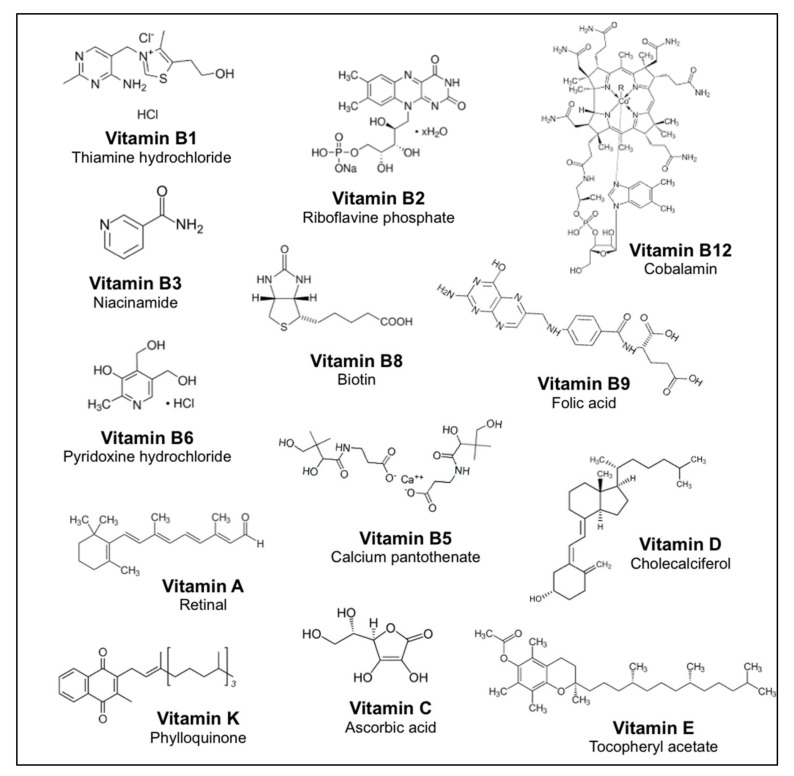
Chemical structure of the thirteen vitamins studied.

Thirteen native or analogous vitamins were selected for this study based on their recurrent use in the nutritional supplement formulation (Figure 1). The following vitamins were provided by Bayer SAS (Global Innovation Center, Gaillard, France): vitamin B1 (thiamine hydrochloride), vitamin B2 (riboflavin phosphate), vitamin B3 (niacinamide), vitamin B5 (calcium pantothenate), vitamin B6 (pyridoxine hydrochloride), vitamin B8 (biotin), vitamin B9 (folic acid), vitamin B12 (cobalamin), vitamin C (ascorbic acid), vitamin A (retinal), vitamin D (cholecalciferol), vitamin K (phylloquinone), and vitamin E (tocopheryl acetate). The compounds were freshly prepared in buffer C1 (130 mM NaCl, 5 mM KCl, 10 mM HEPES, 2 mM CaCl_2_, 10 mM pyruvic acid, pH 7.4, 300 mOsm). Lipophilic compounds such as retinal, cholecalciferol, phylloquinone, and tocopheryl acetate were dissolved in dimethyl sulfoxide (DMSO) at a concentration of 100 mM for the initial solution. To prevent toxic effects on transfected cells or nonspecific cell responses, the final concentration of DMSO in buffer C1 was kept below 0.1% (*v*/*v*) at each concentration for the tested compounds [20]. Dulbecco’s modified Eagle’s medium (DMEM) and all tissue culture media components were purchased from Life Technologies (St Aubin, France).

#### 2.1.2. Expression of TAS2Rs in Heterologous Cells

A functional expression study was carried out as described previously [13,21,22,23]. TAS2R expression vectors (pcDNA5FRT-SST3-TAS2R-HSV and pEAK-10-SST3-TAS2RHSV) were kindly provided by Wolfgang Meyerhof (German Institute of Human Nutrition Postdam-Rehbruecke, Nuthetal, Germany). For all constructs, the N-terminus contained the first 45 amino acids of rat somatostatin 3 (SST3) to improve membrane targeting. A sequence coding for a herpes simplex virus glycoprotein D epitope (HSV-tag) was added at the C-terminus to allow immunocytochemical detection of the receptor [13,14,23,24]. HEK293T cells stably expressing the chimeric G protein α subunit Gα16gust44 [25,26] were seeded into poly-D-lysine-coated 96-well black plates (0.35 × 105 cells/well) in high-glucose DMEM supplemented with 2 mM GlutaMAX, 10% dialysed fetal bovine serum penicillin/streptomycin, and G418 (400 µg/mL), and then placed under a humidified and controlled atmosphere (37 °C and 6.3% CO_2_) overnight. After 24–26 h of incubation, cells were transiently transfected with TAS2R expression plasmids (120 ng/well) using Lipofectamine 2000 reagent (0.4 µL/well) (Thermo Fisher Scientific, Illkirch-Graffenstaden, France).

#### 2.1.3. Calcium Mobilization Assay

Approximately 24–26 h after transfection, cells were loaded for 1 h at 37 °C with Fluo4-AM (2 µM, Molecular Probes), which had been previously mixed with pluronic acid F-127 (0.01%) in C1 buffer (130 mM NaCl, 5 mM KCl, 10 mM HEPES, 2 mM CaCl_2_, 10 mM pyruvic acid, pH 7.4, 300 mOsm) containing 2.5 mM probenecid (Life Technologies). Approximately one hour after loading, the plate was rinsed twice with 100 µL of C1 buffer. The calcium signal was recorded for 90 s after injection of a range of compounds using a Flexstation 3 fluorometric imaging plate reader (Molecular Devices) at a wavelength of 510 nm after excitation at 488 nm. All substance concentrations were measured in triplicate in at least three independent experiments. As cell transfection and substances can interfere with cellular viability and calcium assays, all compounds were tested on mock-transfected cells (cells transfected with empty vectors) on the same plates for all substance concentrations used.

#### 2.1.4. Determination of Half-Maximal Effective Concentrations (EC_50_)

To obtain a dose–response curve, the ratio between the maximum variation in fluorescence after ligand addition and the initial fluorescence before addition (ΔF/F0) was calculated for each well. The fluorescence signals collected for each replicate were averaged for the same substance concentration, and the fluorescence changes in the corresponding mock-transfected cells were subtracted. The dose–response curves were established by plotting the signal amplitude versus log agonist concentration. The dose–response data were fitted, and EC_50_ values were calculated using a four-parameter logistic equation using SigmaPlot software (Systat Software, San Jose, CA, USA).

[f(x) = min + (max − min)/(1 + (x/EC_50_)nH)] (1)

#### 2.1.5. Determination of the Ideal Concentration

The use of high concentrations of fluorescent compounds, such as vitamins B2, B3, and B9, can lead to nonspecific calcium responses throughout TAS2R activation testing. Therefore, all compounds were tested at different concentrations on mock-transfected cells to determine the highest concentration to be used for experiments. For each identified vitamin–receptor pair, a logarithmic concentration range was studied (seven concentrations) from the maximum applicable concentration.

### 2.2. Bitter Taste Detection Threshold in Humans

#### 2.2.1. Stimuli

Based on bibliographic studies, preliminary works performed in the laboratory, and the results of taste receptor functional assays, the following vitamins were included in this study: vitamin B1 (thiamine hydrochloride), vitamin B2 (riboflavin phosphate), vitamin B3 (niacinamide), and vitamin B6 (pyridoxine hydrochloride). These vitamins are present in high quantities in nutritional supplements, safe for humans (except at very high concentrations), and can induce a strong bitter taste [3,7]. The vitamin concentrations used are presented in Table 1. These concentrations were determined according to the toxicity threshold of each compound tested and the maximum daily reference intake. These compounds were prepared in commercial mineral water (Evian^®^, Evian, France).

#### 2.2.2. Three-Alternative Forced Choice (3-AFC)

The threshold values for bitter taste detection were determined by 32 panelists (18–65 years old, 14 males and 18 females, without sensory perception disorders, food allergies, drug treatments, or heart, lung, or cardiac pathologies) who were recruited from Dijon (France) and the surrounding areas. An ethical committee approved this study (Personal Protection Committee of Rennes—Ouest V, protocol code 2018A01342-53), and all panelists signed an informed consent form. A sensory test called three-alternative forced choice (3-AFC) was used in accordance with the protocol detailed in NF ISO 13301. Before performing the measurement sessions, four training sessions were provided to familiarize the panelists with the bitter taste, test procedure, and signal acquisition software. In this discriminative test, six series consisting of three samples, which were each encoded using randomly generated three-digit numbers, were simultaneously delivered to the panelists. The six 3-AFC presentations contained two blank samples (commercial mineral water, manufacturer: Evian, France) and ascending concentrations of vitamin solution. Before the test, subjects were advised that each presentation consisted of two samples that contained only water and one sample that contained a taste. For each 3-AFC presentation, subjects were asked to take the samples sequentially from left to right. Then, they placed the entirety of each sample solution in their mouths and carefully gargled to ensure that the solution was dispersed throughout the mouth. A sip-and-spit procedure was used; subjects rinsed their mouths with commercial mineral water between each series of tests. Subjects were asked to indicate which of the samples contained the stimulus. If a subject perceived no difference between the three samples, he or she was asked to choose one (forced choice). The sensory tests were conducted in a room devoted to sensory evaluation. For the case of colored samples, black-colored glasses were used, and tastings were performed under red light. Preliminary experiments demonstrated that the tested vitamins had olfactory sensory qualities, noticeable by the ortho- and retronasal olfaction. The aim of this sensory analysis was to determine the bitter taste detection threshold for these vitamins and not an olfactory detection threshold. Therefore, the measurements were conducted using pinched nose clips. The subjects waited one minute between each series and had to thoroughly rinse their mouths. Four repetitions were performed per subject for each vitamin to obtain statistically interpretable results, which represented a total of 288 samples. During the sensory analysis session, a bitterness detection threshold of only two vitamins was used (36 samples) to avoid the ingestion of a high vitamin quantity, which is potentially harmful. The answers from the panelists were collected using software dedicated to sensory analysis (FIZZ acquisition V.2.51 software).

#### 2.2.3. Statistical Analysis

The best estimate threshold (BET) method was used to determine the bitter detection threshold for four vitamins [27]. The individual best-estimated detection threshold (BET) was proposed in ASTM E679-04. ASTM E679-04 is a standard practice for the determination of odor and taste thresholds via a forced-choice ascending concentration series method of limits. The taste or odor thresholds were obtained by calculating the geometric mean of the last missed concentration and the next higher concentration detected, which was considered the first subsequent concentration correctly answered. If the subject arrived at the top of the series without correctly identifying the samples or began at the bottom and correctly identified all of them, we extrapolated a value beyond the test series. At the top, this would have been the geometric mean of the highest concentration tested and the next concentration that would have been used in the series if the test had been continued. The same procedure was adopted starting at the bottom. The group of BETs were calculated considering the geometric means of the individual BETs. The data treatment was conducted using statistical analysis software (XLSTAT^®^ 2020.2.3).

## 3. Results

### 3.1. Identification of the TAS2Rs Responding to Vitamins

To identify the bitter taste receptors activated by the 13 selected vitamins (Figure 1), 25 human TAS2Rs were transiently expressed in HEK293T-Gα16gust44 cells, as described previously [13,21,23,28]. Cellular responses were measured using a fluorescent calcium indicator dye after stimulation with varying concentrations of vitamins ranging from moderate to the highest possible concentration determined in pilot experiments. The differences in maximal concentration used are mainly related to vitamin solubility or intrinsic fluorescence properties. This was the case for vitamin B2, which was tested at the lowest concentration (0.01 mM) because of its strong fluorescence. Table 2 shows that four vitamins activate one or two human TAS2Rs, whereas the other vitamins do not activate any other TAS2R-expressing cells even at the highest concentration tested (up to 1–100 mM). Two fat-soluble vitamins, D and A, strongly activate TAS2R10 and TAS2R38 receptors, respectively, at the tested concentration (1 mM). The water-soluble vitamin, B6, elicited a robust response from the TAS2R7 and TAS2R14 receptors when applied at a concentration of 100 mM. Our data also revealed the activation of the TAS2R1 receptor following the addition of 0.1 mM vitamin B1, as previously shown by Meyerhof et al. (2010) [13]. Nevertheless, in our study, we were unable to demonstrate the activation of the TAS2R7 and TAS2R39 receptors. For the TAS2R7 receptor, this difference can be explained by the relatively higher concentrations used in the cellular-based assay compared to our study [19]. For the TAS2R39 receptor, the difference in the chemical structure of the vitamin B1 used, the thiamine hydrochloride in our study, and the thiamine chloride [13] could explain the results obtained.

**Figure 2:**
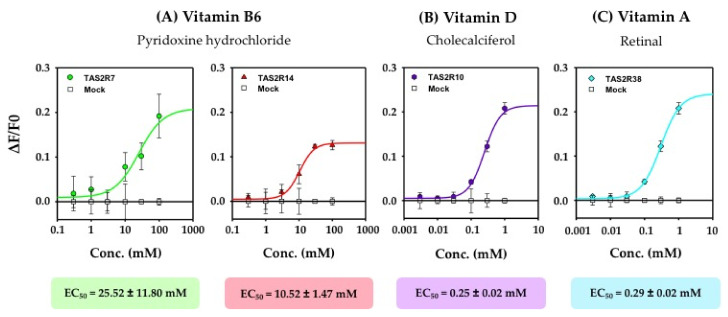
Dose–response curve and EC_50_ value measured with cell lines transiently expressing TAS2Rs stimulated with vitamin B6 (**A**), vitamin D (**B**), and vitamin A (**C**). The EC_50_ value is defined as the concentration required for half-maximal TAS2R activation. Dose–response curves obtained for HEK293 cells transiently transfected with human TAS2Rs in response to vitamins. HEK293 cells expressing TAS2R7, TAS2R10, TAS2R14, and TAS2R38 were stimulated with vitamins B6, D, and A at a concentration range of 0.003–100 mM. Responses of mock-transfected cells to the same concentration of vitamins are also shown as a negative control. Calcium mobilization using Fluo-4 dye was calculated by baseline subtraction of HEK293T cells from the stable cells and plotted as ∆F/F. (**A**–**C**) represent the SEM for three independent experiments performed in triplicate, and error bars represent the standard deviation. EC_50_, half maximal effective concentration; SEM, standard error of the mean; Conc., concentration.

We found that these vitamin responses are dose-dependent (Figure 2). The signal is specific since mock-transfected cells are insensitive to vitamin stimulation. From the dose–response curves, we calculated the half-maximal receptor activation (EC_50_) for each identified vitamin agonist (Figure 2). The two fat-soluble vitamins, A and D, induce calcium responses in TAS2R38- and TAS2R10-expressing cells with EC_50_ values of 0.29 ± 0.20 and 0.25 ± 0.20 mM, respectively. Vitamin B6 activates the TAS2R7 and TAS2R14 receptors with EC_50_ values of 25.52 ± 11.80 and 10.52 ± 1.47 mM, respectively (Figure 2).

Vitamin B1 elicits TAS2R activation only at two concentrations (0.1 mM and 1 mM), and we could not obtain a dose–response curve. However, the lowest concentrations that could elicit TAS2R activation, which is called the cellular bitter taste detection threshold, were calculated (Table 3). The cellular bitter taste detection thresholds for vitamins B1 and B6 are determined to be 0.1 mM and 1 mM, respectively. For vitamins A and D, based on the dose–response curves presented in Figure 2, a detection threshold value for cellular bitter taste is determined to range between 10 and 30 µM. According to their lower EC_50_ values and cellular bitter taste thresholds, vitamins A and D are revealed to be the most potent bitter compounds.

### 3.2. Human Bitter Taste Detection Threshold of Vitamins

The cellular assay utilized here can be used to detect receptor activation using a calcium-sensitive fluorescent dye. Therefore, this method cannot be applied to measure receptor activation by high concentrations of fluorescent compounds, such as vitamins B2 or B3, whose bitterness was determined by a sensory panel. Therefore, we conducted human sensory experiments to determine their bitter taste detection thresholds and evaluate their real contribution to the nutritional supplement bitterness. The human bitter taste detection threshold corresponds to the minimum vitamin concentration that causes sufficient TAS2R activation for the perception of bitterness. In addition, to compare cellular and human data, two other vitamins previously identified as bitter in the taste receptor functional assay performed in this study were included in the sensory analysis: vitamin B1 (thiamine hydrochloride) and vitamin B6 (pyridoxine hydrochloride). Fat-soluble vitamins (A and D), for which bitterness has been previously revealed by the same cellular-based assay, were not included in this sensory analysis for human safety reasons related to their high concentrations [29].

**Figure 3:**
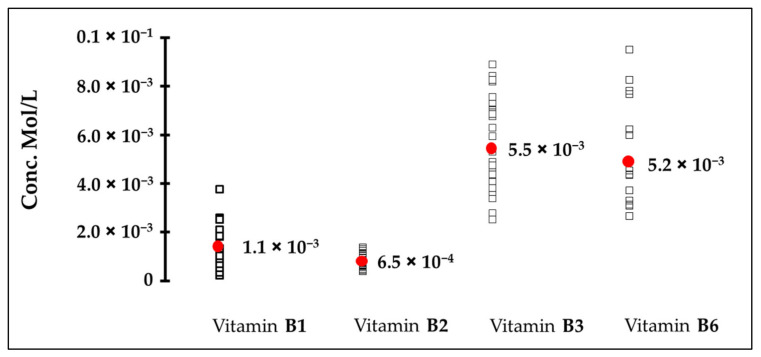
Human bitter taste detection threshold (mol/L) of four water-soluble vitamins.

The bitter detection thresholds for the four tested vitamins are shown in Figure 3. The results confirm the bitterness of vitamin B1 (thiamine hydrochloride) and vitamin B6 (pyridoxine hydrochloride), already identified in the receptor cellular assay, with bitter taste detection thresholds of 1.1 mM and 5.2 mM, respectively. This sensory experiment also demonstrates and confirms the bitterness of vitamins B2 and B3, which was not identified in the cellular-based assay, with bitter taste detection thresholds of 0.65 mM and 5.5 mM, respectively. With a lower bitter taste detection threshold value, vitamin B2 shows a higher activation potency than the other vitamins tested (B1, B3, and B6).

## 4. Discussion

The bitterness of vitamins is an important issue, since it can largely affect a patient’s acceptance of nutritional supplements and/or supplemented food. The aim of the present study was to determine whether the selected vitamins could be used to activate human bitter taste receptors. After identification of the activated receptors, dose–response curves for each agonist receptor pair were generated, and the EC_50_ values were determined. Although various studies have been conducted to highlight the sensory characteristics of vitamins, no data are available for their taste detection threshold, especially bitter taste. To support the validity of the observations measured in the cellular-based assay, we determined the bitter taste detection threshold for four vitamins that exhibit a strongly bitter taste using sensory analysis.

### 4.1. Comparison of Cellular-Based Assay and Sensory Analysis

The cellular-based assay demonstrated the bitterness of four vitamins: B1 (thiamine hydrochloride), B6 (pyridoxine hydrochloride), A (retinal), and D (cholecalciferol), and revealed for the first time the involvement of TAS2R receptors in the bitterness detection of vitamins B6, A, and D. Comparing the EC_50_ values obtained for each vitamin identified as bitter, we can reveal that the two fat-soluble vitamins (D and A) have a higher activation potency than that of the water-soluble vitamin (B6). This difference is less pronounced for the activation efficacy, with relatively similar maximal amplitude values for all tested compounds.

Surprisingly, vitamin D is found to activate TAS2R10, while it is described as tasteless by sensory analysis [7]. However, it should be noted that the vitamin D concentration is omitted in this paper. Thus, one explanation is that the observed activation of TAS2R10 could be related to the concentration range tested in the cellular-based assay, which could be higher than the concentrations used in the sensory study.

As previously observed, cellular data do not perfectly correlate with human oral detection mechanisms. The molar concentration for each previously identified bitter vitamin (determined for a standard nutritional supplement and according to the recommendations of pharmaceutical companies) and associated bitter taste detection thresholds (determined from cellular and human experiments) are presented in Table 3. The cellular bitter taste detection threshold values are lower than the values obtained by the sensory analysis for vitamins B6 (pyridoxine hydrochloride) and B1 (thiamine hydrochloride). This observation can be explained by two possible phenomena that remain to be explored: The cellular-based assays are performed in buffer media in which the composition is different from that of saliva. As a consequence, this can lead to a change in the availability of tastants for the receptors [30] or a change in their potential participation in signal activation [31]. In addition, absorption by the oral epithelium and/or interaction with salivary proteins can result in differences between human bitterness perception and TAS2R activation in cellular-based assays [16,30]. Another explanation can be drawn at the central level: Flavor perception is a cerebral construction resulting from the integration of chemosensory signals in the brain arising from ingredient ingestion. Regarding the perception of taste, there are several stages in the brain information treatment provided by neural signals, including external signals that can interact with taste perception [31]. These results also suggest that a dilution phenomenon for the sample by saliva could be responsible for an increase in the bitter taste detection threshold in humans. Therefore, these results highlight that the cellular-based assay enables a rapid screening of a large number of molecules to reveal their potential bitterness and the identity of the activated TAS2Rs. However, this technique does not allow for the identification of all bitter vitamins. For instance, sensory analysis reveals the bitterness of two vitamins, B2 (riboflavin phosphate) and B3 (niacinamide), while no information was obtained using cellular-based assays due to their intrinsic fluorescence properties at high concentrations.

### 4.2. Relationship between the Vitamin Functions and TAS2R Activation

TAS2Rs have been observed in many extraoral tissues, revealing that their physiological function is not limited to sensory oral perception [32,33]. Recent studies have shown that these extraoral receptors are involved in many physiological processes [34]. In this study, we demonstrate that vitamin D (cholecalciferol) and vitamin B6 (pyridoxine hydrochloride) activate TAS2R10 and TAS2R14 receptors, respectively. Interestingly, these two broadly tuned TAS2Rs are also expressed in different extraoral tissues, such as the smooth muscles of different organs for TAS2R10 or the brain for TAS2R14 [35,36,37]. In addition to being bitter molecules, vitamin D (cholecalciferol) and vitamin B6 (pyridoxine hydrochloride) are considered active compounds involved in different physiological human body functions. For example, vitamin D acts on muscular contraction mechanisms, and vitamin B6 is essential for brain development [38,39]. Our data suggest a link between TAS2R10 and TAS2R14 extraoral localization and the targeted site for the action of these two vitamins (D and B6). In other words, these data suggest that the physiological action of vitamin D in smooth muscles and vitamin B6 in the brain can be partly linked to TAS2R receptor (TAS2R10 and TAS2R14) activation.

Interestingly, we find that vitamin A (retinal) activates TAS2R38, a receptor specialized in the recognition of a particular molecular pattern (S–N=C) of isothiocyanates. As retinal (vitamin A) lacks this S–N=C pattern, this observation clearly demonstrates the participation of TAS2R38 in the detection of bitter molecules other than isothiocyanates. The TAS2R38 receptor has also been previously identified to exist in extraoral tissues, such as upper airway tissues, where it is involved in the detection of chemical molecules produced by bacteria [35].

### 4.3. Vitamins, Nutritional Supplements, and Bitter Taste

Nutritional supplements contain different amounts of nutrients, such as vitamins. From the data obtained from the cellular assay and sensory analysis, we can identify six bitter vitamins at different concentrations relative to their physicochemical properties. Therefore, these vitamins can cause an unpleasant aftertaste that appears a few seconds after consumption of a nutritional supplement. Comparing the bitter taste detection threshold obtained in both experiments with the calculated concentrations in standard nutritional supplements (Berocca^®^, Bayer France), there is no evidence that vitamins have a negative impact on these organoleptic qualities (Table 3). For each bitter vitamin identified previously, the cellular and human bitter taste detection threshold values are lower than the concentrations encountered in the reference nutritional supplement. However, these results should not preclude the vitamin impact on the sensory characteristics of nutritional supplements. As already described, additive effects between bitter nutrients such as minerals or amino acids could be responsible for the bitterness of nutritional supplements [3,40]. As the TAS2R(s) responsible for vitamin B2 (riboflavin phosphate) and vitamin B3 (niacinamide) detection could not be identified, this additive effect at the receptor level should be taken into consideration [16].

## 5. Conclusions

Our results suggest the participation of several TAS2Rs in the detection of bitter vitamins. We measured the human bitter taste detection threshold for four vitamins in aqueous solution, vitamin B1 (thiamine hydrochloride), vitamin B2 (riboflavin phosphate), vitamin B3 (niacinamide), and vitamin B6 (pyridoxine hydrochloride), which exhibit a strong bitter taste. A combination of in vivo sensory and in vitro biological analyses suggests that the bitter taste perception of vitamins can be explained by the interaction with TAS2R receptors. This conclusion provides precise information for the sensory attributes of vitamins, mainly bitterness.

## Figures and Tables

**Table 1:** Vitamin concentrations used (mM) for the human bitter taste detection threshold.

Vitamins	Concentration (mM)					
	C1	C2	C3	C4	C5	C6
Thiamine hydrochloride (B1)	0.22	0.45	0.89	1.78	3.56	7.12
Riboflavin phosphate (B2)	0.16	0.24	0.37	0.55	0.83	1.25
Niacinamide (B3)	2.03	3.05	4.57	6.85	10.30	15.40
Pyridoxine hydrochloride (B6)	0.24	0.60	1.52	3.80	9.50	23.70

**Table 2:** Response profiles for 25 TAS2Rs stimulated with 13 vitamins and their maximal applicable concentration (M.A.C) in the receptor assay. Orphan receptors (TAS2R19, 42, 45, and 60) are not shown.

Vitamin	M.A.C (mM)	TAS2R Receptor																				
		1	3	4	5	7	8	10	13	14	16	20	30	31	38	39	39	40	41	43	46	50
B1	1	**+**																				
B2	0.01																					
B3	3																					
B5	30																					
B6	100					**+**				**+**												
B8	3																					
B9	3																					
B12	3																					
C	10																					
A	1														**+**							
E	1																					
K	1																					
D	1							**+**														

**+**: positive responses (lines with background color).

**Table 3:** Comparison between the cellular bitter taste detection threshold, human bitter taste detection threshold, and concentration encountered in nutritional supplements studied for six bitter vitamins.

Vitamin	Cellular Bitter Taste Detection Threshold (mM)	Human Bitter Taste Detection Threshold (mM)	* Concentration in Nutritional Supplement (mM)
B1	0.10	1.10	0.44
B2	ND	0.65	0.40
B3	ND	5.50	4.06
B6	1.00	5.20	0.73
A	0.05	ND	0.03
D	0.05	ND	0.01 × 10^−5^

* Calculated molar concentration for a nutritional supplement (Berocca^®^, Bayer France) dissolved in 100 mL of water. ND, not determined.

## Data Availability

Not applicable.
